# Basaloid Squamous Cell Carcinoma of the Dorsum of the Tongue Following Chronic Hypertrophic Candidiasis: A Case Report and Literature Review

**DOI:** 10.7759/cureus.82951

**Published:** 2025-04-24

**Authors:** Shiori Kyoda, Manabu Yamada, Kenichiro Suga, Masafumi Nishikawa, Seiji Asoda

**Affiliations:** 1 Department of Dentistry and Oral Surgery, National Hospital Organization (NHO) Tochigi Medical Center, Tochigi, JPN; 2 Department of Pathology, National Hospital Organization (NHO) Tochigi Medical Center, Tochigi, JPN; 3 Department of Dentistry and Oral Surgery, Keio University School of Medicine, Tokyo, JPN

**Keywords:** alcohol consumption, basaloid squamous cell carcinoma, chronic hyperplastic candidiasis, dorsum of the tongue, oral cancer

## Abstract

Basaloid squamous cell carcinoma (BSCC) is a rare and aggressive subtype of squamous cell carcinoma (SCC). The prognosis of patients with BSCC is worse than that of patients with normal SCC. Chronic hyperplastic oral candidiasis (CHC) is characterized by proliferative changes in the epithelial tissues and is associated with a high risk of carcinogenesis. We herein report a case of BSCC of the dorsum of the tongue following CHC. The patient was a 64-year-old man; *Candida albicans* was detected on the dorsal surface of his tongue in 2007. He was followed at our hospital under a diagnosis of CHC. In 2022, a 35×20 mm area of erythematous mucosa surrounded by white spots and induration was found in the same region, and a biopsy revealed a diagnosis of poorly differentiated SCC. After combination treatment with paclitaxel, carboplatin, and cetuximab (PCE) induction chemotherapy (PCE), the patient underwent neck dissection, hemiglossectomy, and oral reconstruction using an anterolateral thigh skin flap under general anesthesia. The final diagnosis was BSCC, and there was no evidence of recurrence or metastasis at 23 months postoperatively. To our knowledge, this is the first report of a case of BSCC following tongue CHC. In this case, chronic inflammation caused by CHC, chronic alcohol consumption, and diabetes mellitus may have been associated with the development of BSCC. Considering the potential role of CHC in the development of malignant diseases, careful and continuous follow-up is required.

## Introduction

Basaloid squamous cell carcinoma (BSCC) is a subtype of squamous cell carcinoma (SCC) first described by Wain et al. in 1986 [1]. BSCC is associated with the development of early cervical lymph node metastasis and distant metastasis, is clinically more aggressive, and has a worse prognosis than conventional SCC [2]. BSCC is mainly found in the upper gastrointestinal tract, larynx, mid-pharynx, hypopharynx, thymus, and cervix, while cases involving the oral cavity are relatively rare [3, 4]. Chronic candidiasis has long been suggested to be associated with oral cancer [5] and was added as an oral potentially malignant disorder (OPMD) in the fourth edition of the WHO's Classification of Head and Neck Tumours in 2017, although it was re-excluded in the fifth edition in 2022. Chronic hyperplastic candidiasis (CHC) is characterized by proliferative changes in epithelial tissues and is considered to be associated with a higher risk of carcinogenesis [5,6]. However, to the best of our knowledge, there have been no reports of BSCC arising from chronic candidiasis, including CHC. We herein report a case of BSCC of the tongue that occurred after CHC of the tongue and review the relevant literature on this rare entity.

## Case presentation

**Figure 1:**
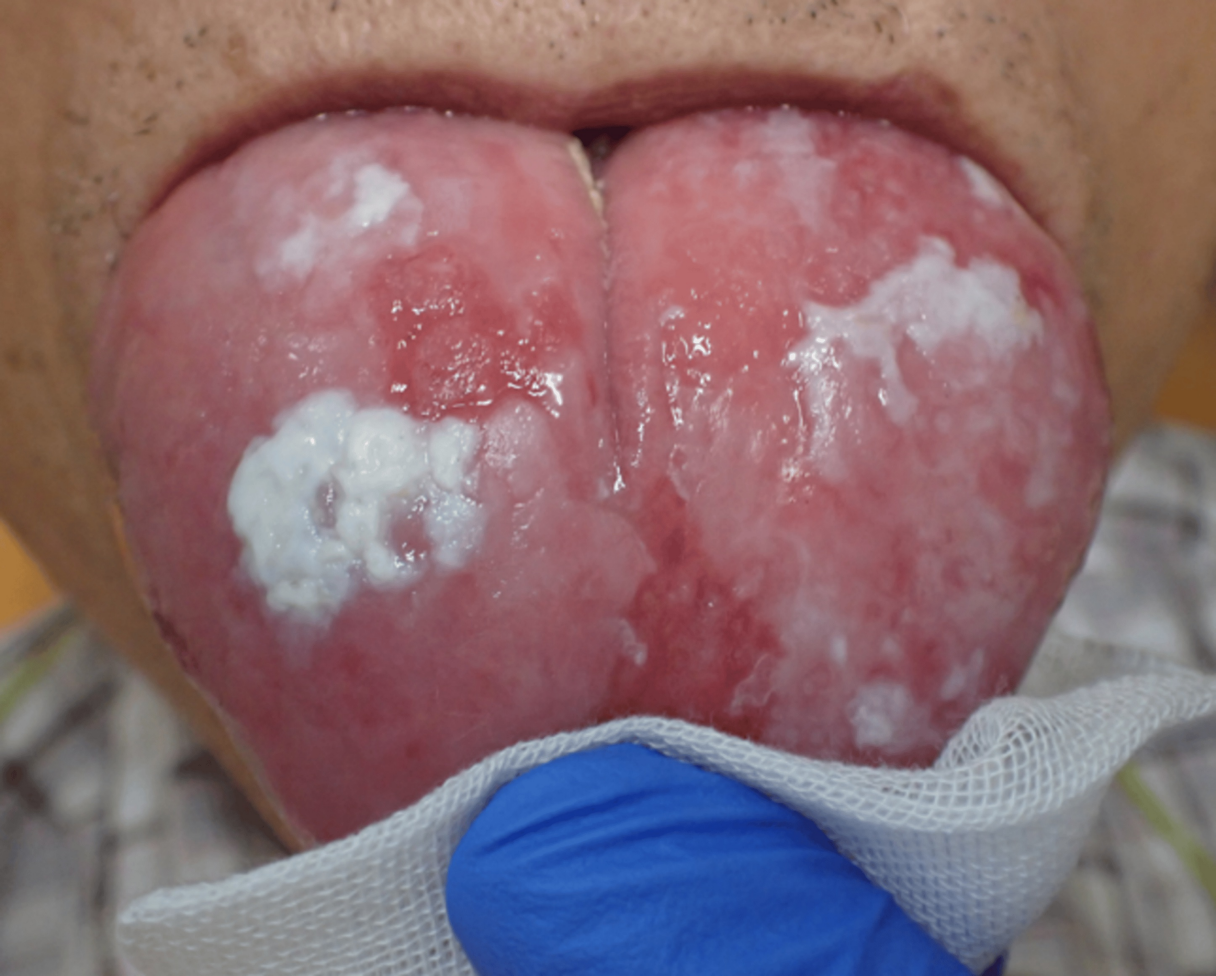
Intraoral photographs obtained in September 2020 An erythematous white lesion of 35 mm in length was observed on the dorsum of the tongue.

**Figure 2:**
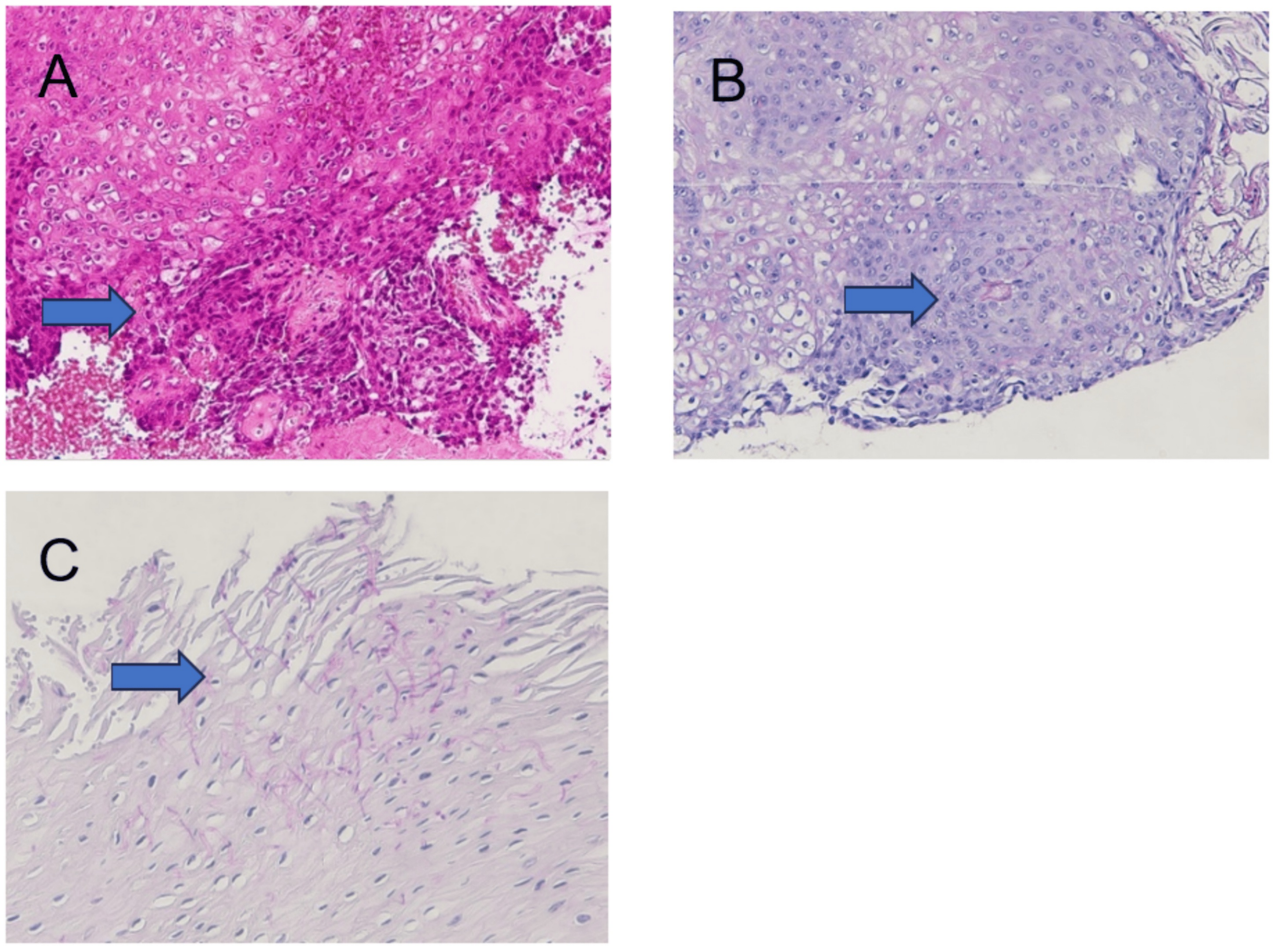
Histopathological findings at the biopsy in September 2020 Heteromorphic cells with a high nuclear-to-cytoplasmic (N/C) ratio (arrow) (A, hematoxylin-eosin staining, ×40) were observed, and many *Candida *organisms were observed in the keratinocytes (arrow) (B, periodic acid-Schiff staining, ×40). In addition, numerous *Candida *organisms were observed within the epithelium (arrow) (C, periodic acid-Schiff staining, ×40).

The patient was a 64-year-old man; *Candida albicans* was detected on the dorsal surface of his tongue in 2007. He received continuous follow-up at our hospital and was diagnosed with CHC in the same region. The patient had a history of diabetes mellitus and hyperlipidemia, but there was nothing to note in his family history. The patient had no history of smoking; however, he had a 44-year history of alcohol consumption. In September 2020, a 35-mm erythema was observed on the dorsum of the tongue, raising the suspicion of malignant transformation (Figure 1). The mucosa along the lateral border of his tongue appeared clinically unremarkable, with no evidence of impaired tongue mobility. Therefore, a biopsy was performed at the site, which revealed a diagnosis of dysplastic squamous epithelium with *Candida* infection (Figure 2).

**Figure 3:**
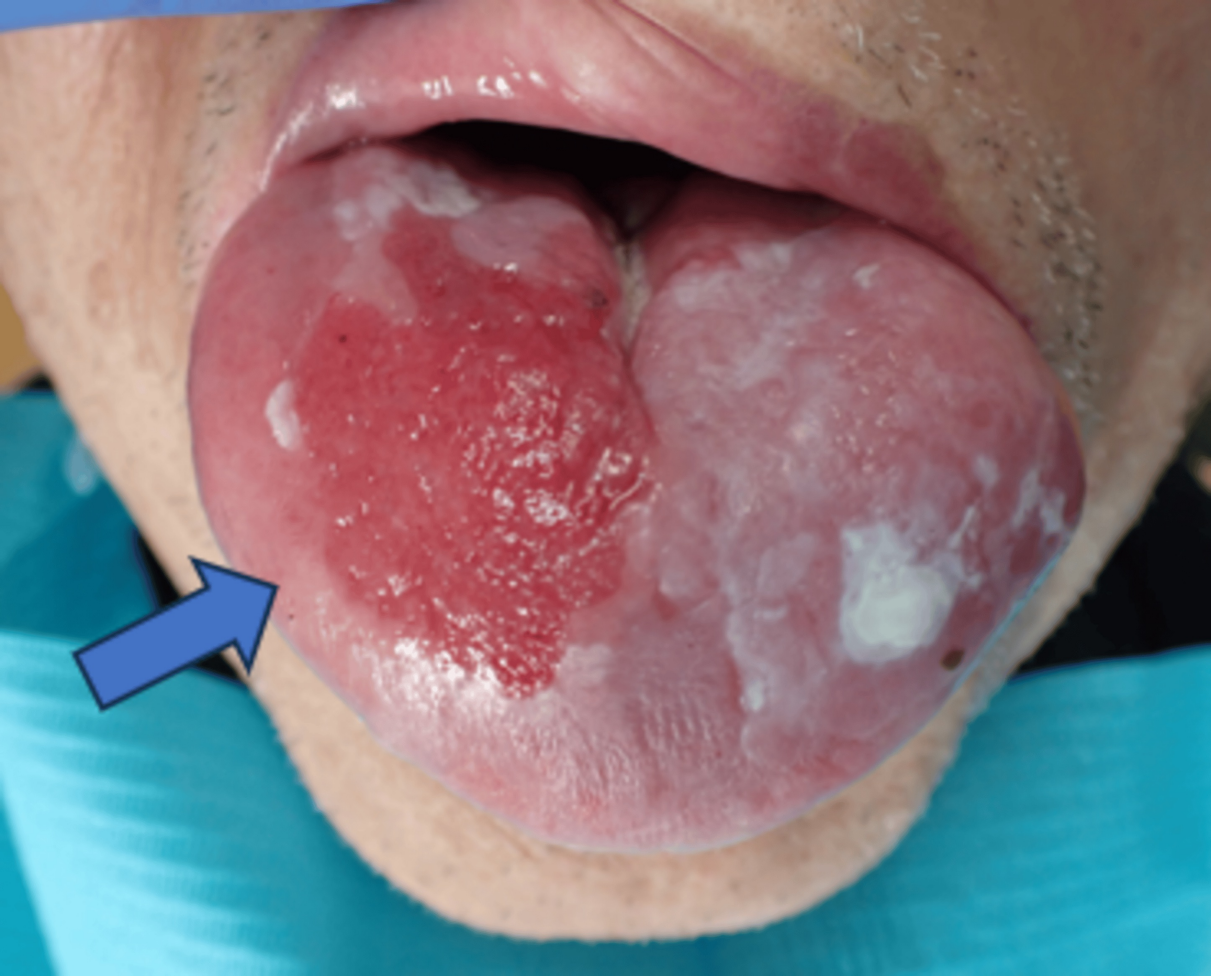
Intraoral photographs at the time of the second biopsy in January 2023 The redness on the right dorsal tongue is enlarged (arrow).

**Figure 4:**
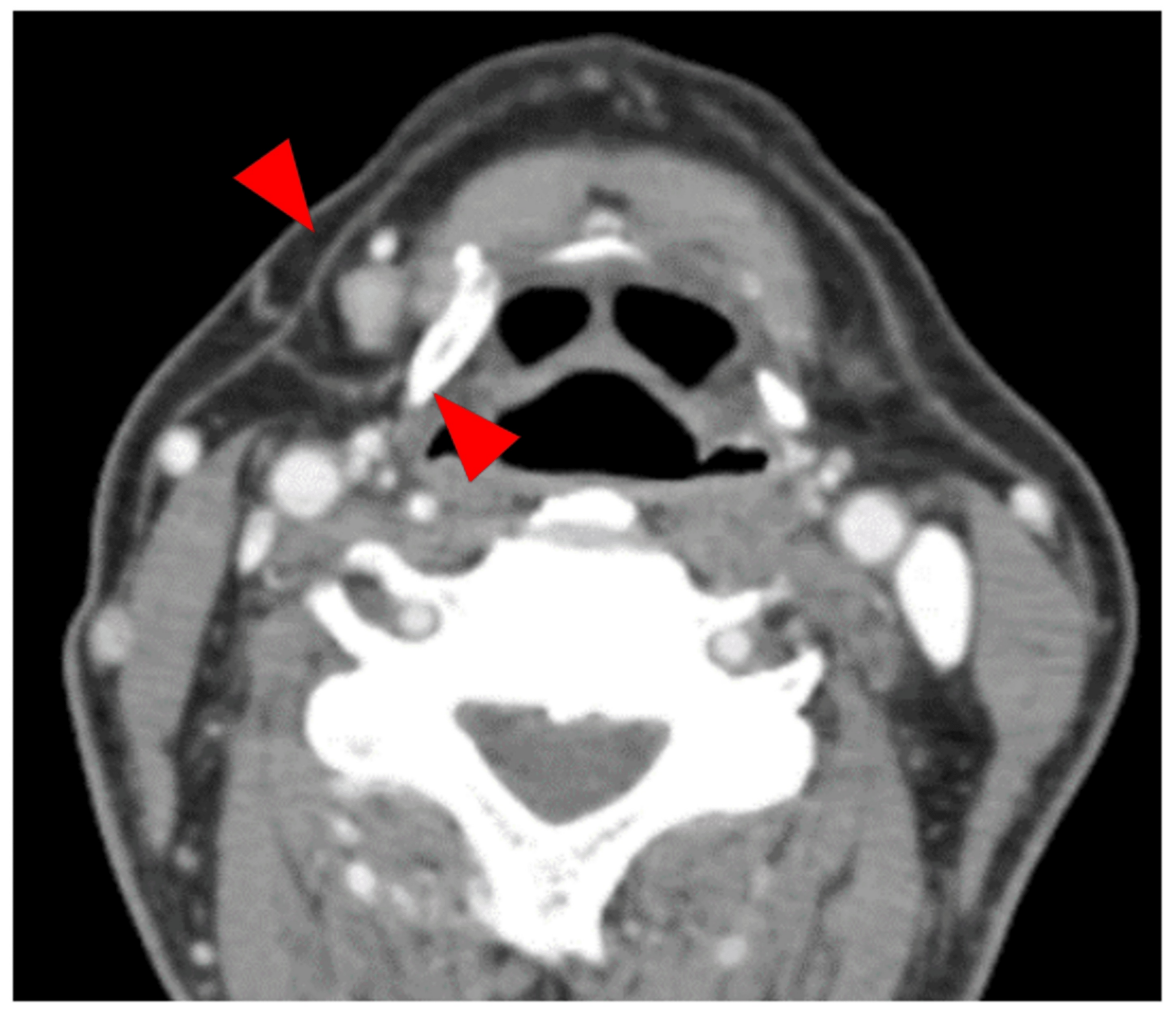
Contrast-enhanced computed tomography findings A swollen lymph node measuring 9 mm in short-axis diameter is observed in the right IB region (arrow). IB: submandibular group of lymph nodes

**Figure 5:**
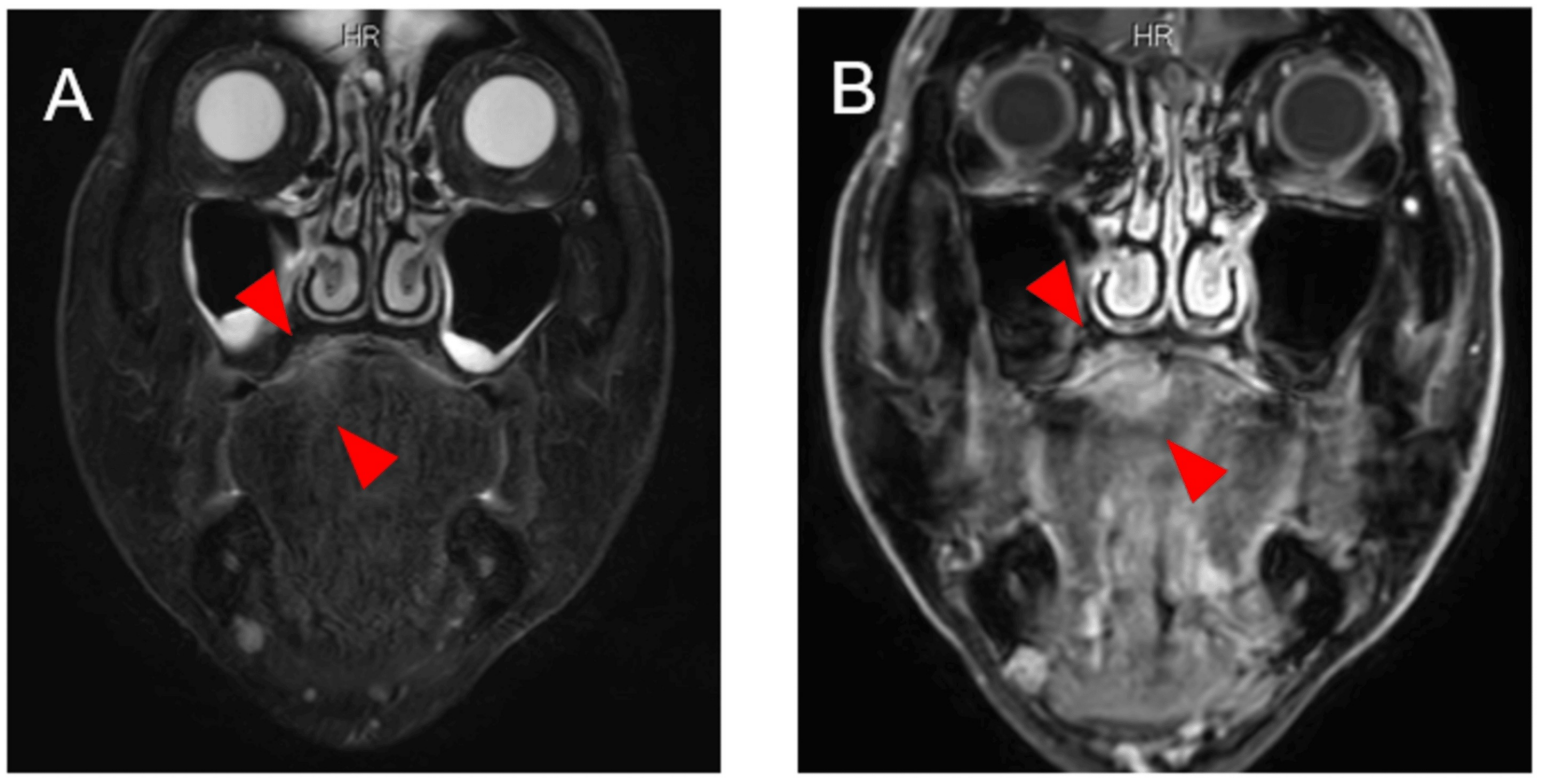
Magnetic resonance imaging findings (A) Fat-suppressed T2-weighted images showing a borderline indistinct lesion with moderate signal intensity on the right dorsal tongue (arrow); (B) T1-weighted images with gadolinium enhancement show a slightly ill-defined enhancement contrast effect on the right dorsal tongue (arrow).

**Figure 6:**
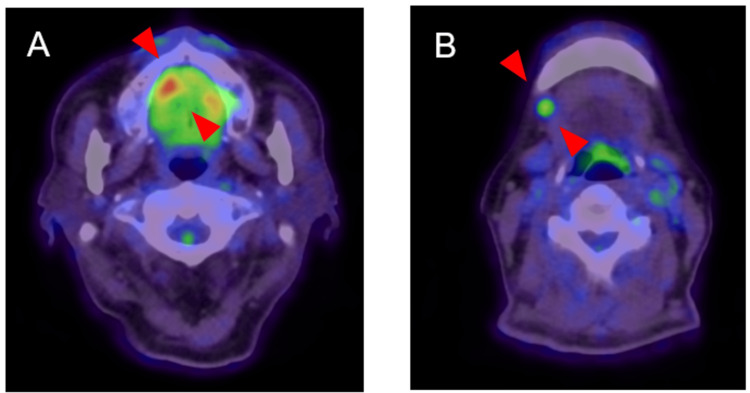
Positron emission tomography/computed tomography findings The primary (A) and right cervical level IB lymph nodes (B) had a maximum standard uptake value (SUV max) of 6.78 and 3.64 uptake, respectively (arrows). IB: submandibular group of lymph nodes

Subsequently, follow-up was continued, but the erythema on the dorsum of the tongue became enlarged in December 2022. In January 2023, when the patient was 79 years of age, a re-biopsy was performed at the same site, and a histopathological examination revealed poorly differentiated SCC. The general findings were normal, and a 35 × 20 mm erythema with mild induration, surrounded by leukoplakia, was observed on the right dorsal tongue (Figure 3). Contrast-enhanced computed tomography (CECT) showed 9 mm lymphadenopathy in the submandibular group of lymph nodes (level IB region) on the right side of the neck (Figure 4). Magnetic resonance imaging (MRI) showed moderate signal intensity on fat-suppressed T2-weighted images, and T1-weighted images with gadolinium enhancement showed contrast enhancement in the right dorsal tongue, with a poorly defined lesion. However, no invasion of extrinsic tongue muscles was observed (Figure 5). Positron emission tomography/CT (PET/CT) showed maximum standard uptake value (SUV max) values of 6.78 and 3.64 in the primary and right cervical level IB lymph nodes, respectively, with no abnormalities in other organs such as the lungs (Figure 6). Ultrasonography and fine-needle aspiration (FNA) cytology of the neck revealed poorly differentiated SCC in the right submandibular lymph node. Based on these findings, the patient was diagnosed with right-sided tongue SCC (cT2N1M0, Stage III).

**Figure 7:**
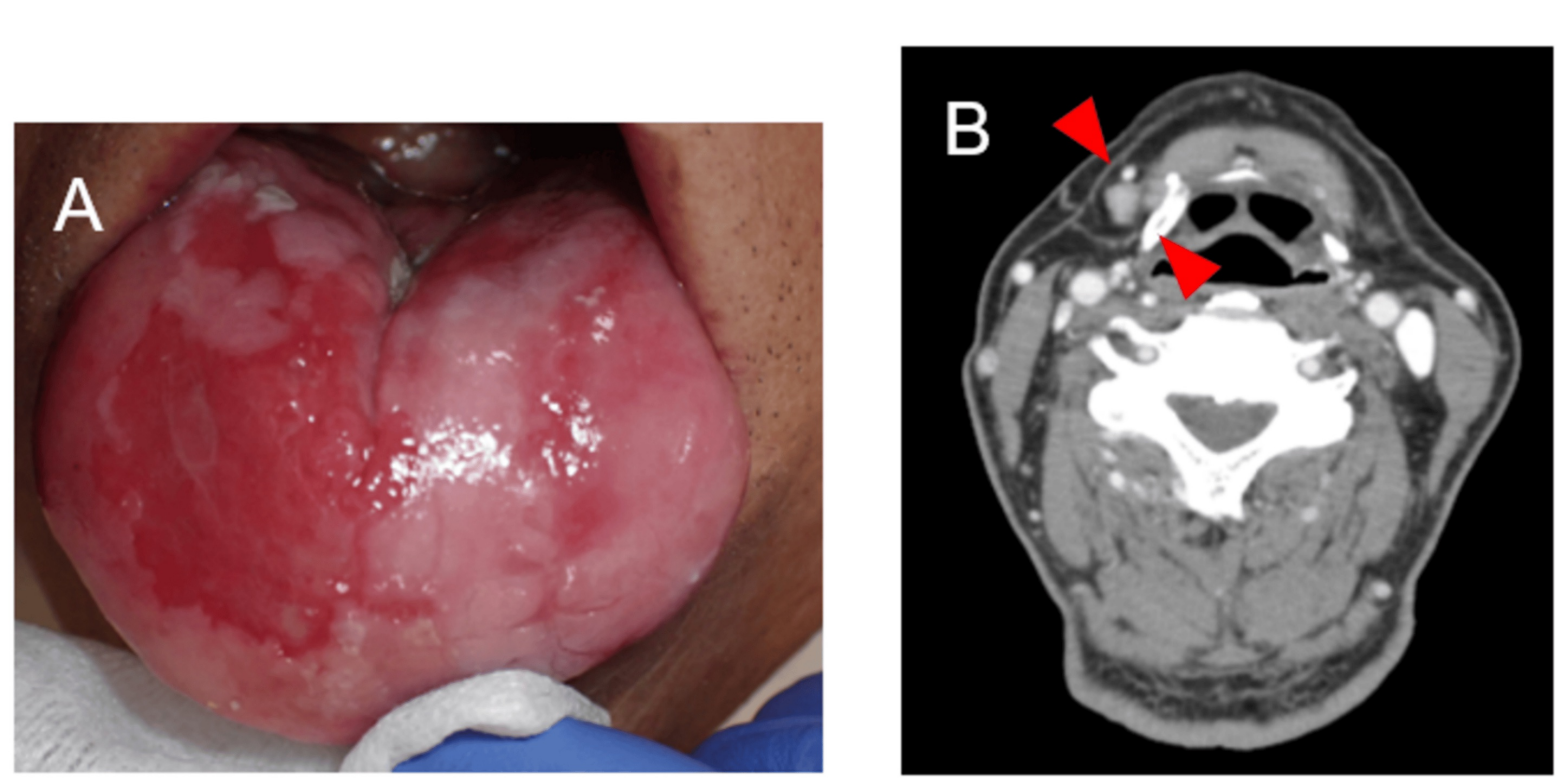
Findings after induction chemotherapy (A) Intraoral radiograph showing no obvious tumor shrinkage; (B) Contrast-enhanced computed tomography reveals no change in the size of the right IB lymph node (arrow) IB: submandibular group of lymph nodes

**Figure 8:**
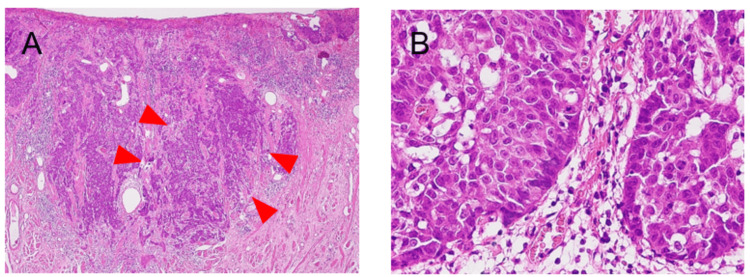
Pathohistological findings of the resected specimen (hematoxylin-eosin staining) (A) The tumor infiltrated the superficial layer of the muscularis, with tumor cells arranged in nests and cords (arrow) (×10); (B) Solid proliferation of basaloid atypical cells with a high nuclear-to-cytoplasmic (N/C) ratio was observed. The nuclei were hyperchromatic, and peripheral palisading of nuclei was noted at the margins of the cell nests (arrow) (×40).

**Figure 9:**
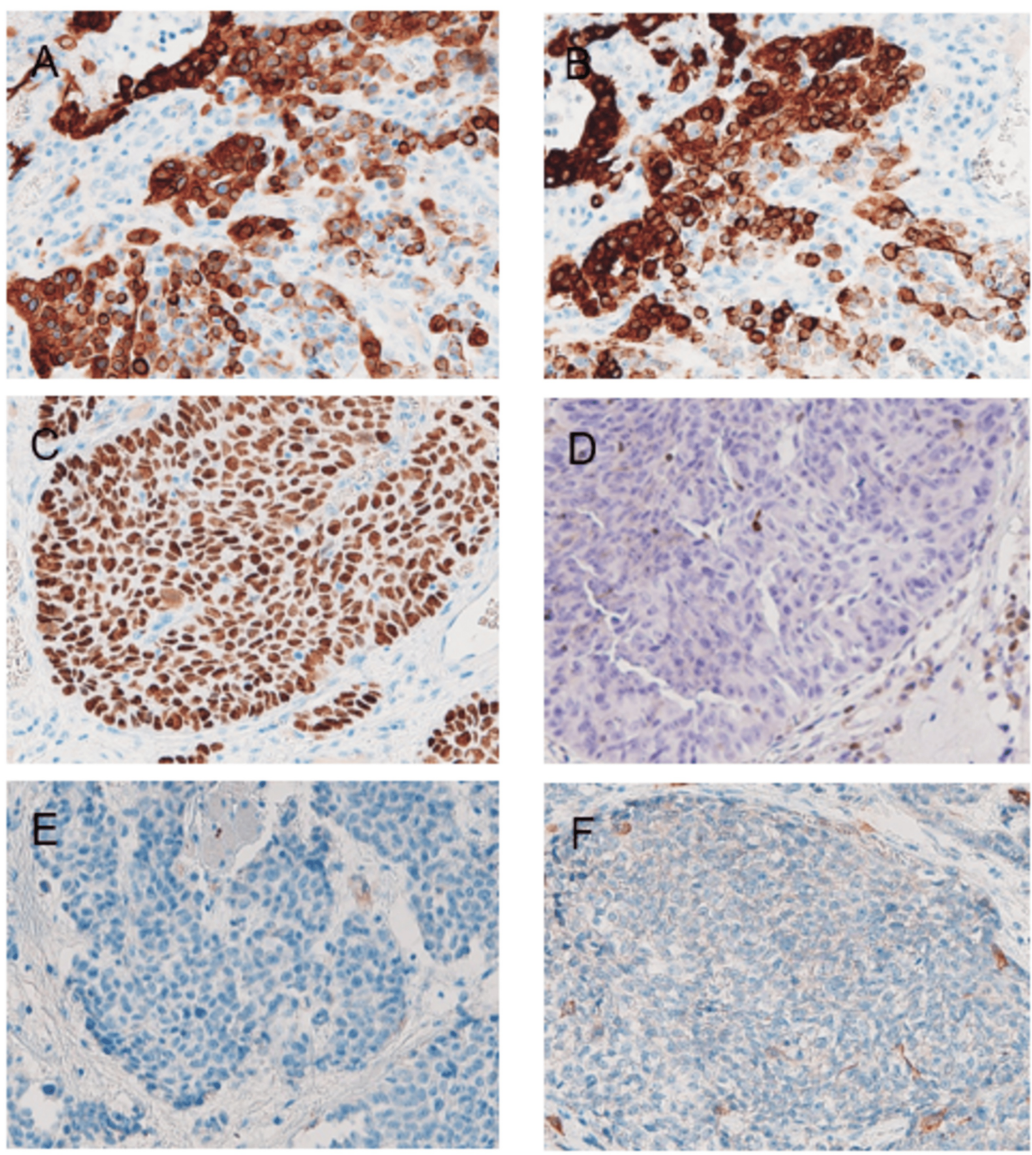
Immunohistological findings of the primary lesion (40) Tumor cells were positive for 34βE12 (A), CK5/6 (B), and p63 (C), and negative for bcl-2 (D), calponin (E), and CD117 (F).

The initial treatment consisted of four courses of paclitaxel, carboplatin, and cetuximab (PCE) induction chemotherapy (ICT) starting in March 2023 (paclitaxel (80 mg/m²), carboplatin (area under the curve (AUC) 1.5), and cetuximab (400 mg/m², initial dose followed by 250 mg/m²)) once weekly. The patient was diagnosed with neutropenia (grade 3) after two courses, followed by two courses of cetuximab monotherapy. The treatment response after ICT, according to the Response Evaluation Criteria in Solid Tumors (RECIST 1.1), was stable disease (SD) (Figure 7). Subsequently, in April 2023, the patient underwent tracheostomy, right extended right supraomohyoid neck dissection involving levels I to IV, hemiglossectomy, and oral reconstruction using an anterolateral thigh skin flap under general anesthesia. The resected specimen was an endophytic lesion measuring 30 × 23 mm. A histopathological examination with hematoxylin-eosin staining revealed tumor invasion from the subepithelium to the shallow muscular layer. Basal cell-like atypical cells were mainly arranged in island-like and cord-like arrangements, and fenestrated nuclei were observed at the margins of cell nests in some places. The cells had a high nuclear-to-cytoplasmic (N/C) ratio, darkly stained nuclei, and enlarged nucleoli (Figure 8). Immunostaining was positive for high-molecular-weight CK (34βE12), CK5/6, and p63, but negative for bcl-2, calponin, and CD117 (Figure 9). On the basis of these findings, the final diagnosis was BSCC. The resection margins of the primary lesion were negative, and lymphatic invasion was observed, although venous invasion was not observed. Metastatic lymph nodes were identified in one node in level IB, three nodes in level IIA, and one node in level IIB, and the pathological stage was stage IVA (ypT2N2bM0). Although no extranodal extension was observed in the metastatic lymph nodes, BSCC has a high rate of distant metastasis and recurrence [4], and postoperative adjuvant chemoradiotherapy was planned. However, the patient declined treatment; therefore, treatment was not performed. Postoperatively, candidiasis of the dorsum of the tongue was detected several times but responded well to oral itraconazole therapy. Twenty-three months after surgery, there has been no recurrence, and the patient is doing well.

## Discussion

BSCC in the head and neck region has been positioned as a subtype of SCC since the second edition of the WHO histological classification in 1991 [7], with a predilection for the larynx and pharynx, and is found scattered in the base of the tongue, floor of the mouth, and larynx [3, 8-11]. The frequency is as low as 2% to 5% among all head and neck cancers, and it is most frequently reported in men between 60 and 70 years of age [12]. To our knowledge, 33 cases of BSCC of the tongue have been reported since 1991 [8, 13-26], with a male-to-female patient ratio of approximately 8:3 and a mean age of approximately 60 years, which is generally consistent with previous reports (Table 1). Clinically, BSCC often presents as indurated, verrucous, painless masses or ulcers and is difficult to distinguish from SCC by visual inspection and palpation alone. Of the eight 33 patients with preoperative biopsy results, seven were diagnosed with BSCC (87.5%). Histologically, it shows mixed histology with a basal cell-like component resembling adenoid cystic carcinoma and an SCC component [1, 27-29]. Of the 33 patients mentioned earlier, 12 had a history of smoking, and eight (66.6%) were current smokers. Nine of the 33 patients had a history of alcohol consumption, and eight (88.8%) reported alcohol use. The patient did not smoke but had a long history of alcohol consumption.

**Table 1:** Reported cases of BSCC of the tongue since 1991 M: male, F: female; ACC: adenoid cystic carcinoma; BSCC: basaloid squamous cell carcinoma; S: surgery; RT: radiation therapy; CCRT: concurrent chemotherapy; CT: chemotherapy; ND: neck dissection; NA: not available; NED: no evidence of disease; DOD: dead of disease; AWD: alive with disease; Y: years; M: months

First author/reference	Year	Age (years)/Sex	Location of the lesion	Alchol	Smoking	Biopsy result	TNM	Stage	Treatment (Primary treatment→Secondary treatment)	Follow-Up
Raslan et al. [13]	1994	65/M	Base of the tongue	NA	NA	NA	T2N0Mx	NA	S with ND	NED, 2Y
		65/M	Base of the tongue	NA	NA	NA	T4N0M0	NA	S with ND→CT	NED, 17M
		56/M	Base of the tongue	NA	NA	NA	T2N2M1	NA	S with ND	DOD, 4M
		64/F	Base of the tongue	NA	NA	NA	T2N0M0	NA	S with ND + RT	DOD, 42M
		75/M	Base of the tongue	NA	NA	NA	T1N1Mx	NA	S with ND	NED, 18M
Ide et al. [14]	1996	72/F	Oral tongue	NA	NA	BSCC	NA	NA	S	NED, 2Y
Altavilla et al. [15]	1999	57/M	Oral tongue	NA	NA	NA	NA	III	S with ND + RT	NED, 10M
Ereño et al. [16]	2008	54/M	Oral tongue	＋	NA	NA	NA	IV	NA	DOD, 18M
Paulino et al. [17]	2009	56/M	Oral tongue	＋	－	NA	T2N0M0	NA	CT	NED, 2Y
		49/M	Oral tongue	＋	＋	NA	T4N0M0	NA	S + CCRT	NED, 33M
		80/M	Oral tongue	NA	NA	NA	T2N0M0	NA	S	DOD, 2M
Matsui et al. [18]	2011	76/M	Oral tongue	NA	NA	BSCC	T2N0M0	NA	CCRT＋S with ND	AWD, 4Y
Heera et al. [19]	2016	54/M	Oral tongue	NA	＋	BSCC	NA	NA	NA	NA
Kumari et al. [20]	2017	39/M	Oral tongue	NA	＋	BSCC	NA	NA	S	NA
Kusafuka et al. [21]	2020	81/M	Oral tongue	NA	NA	ACC	NA	NA	S with ND	NED, 1Y
Schuch et al. [22]	2020	56/M	Oral tongue	－	－	NA	NA	NA	NA	NA
		59/M	Oral tongue	＋	＋	NA	NA	NA	NA	NA
		72/M	Oral tongue	＋	＋	NA	NA	NA	NA	NA
		53/M	Oral tongue, tonsils	＋	＋	NA	NA	NA	NA	NA
Hicks et al. [23]	2020	NA	Oral tongue	NA	NA	NA	T2N1M0	III	S with ND	NED, 7Y
		NA	Oral tongue	NA	NA	NA	T3N0M0	III	S with ND	NED, 15Y
		NA	Oral tongue	NA	NA	NA	T3N0M0	III	S with ND	NED, 11Y
		NA	Oral tongue	NA	NA	NA	T1N0M0	I	S	NED, 36Y
Zhou et al. [24]	2021	71/M	Oral tongue	NA	NA	NA	NA	II	NA	AWD, 11Y
		52/M	Floor of the mouth, tongue	NA	NA	NA	NA	IV	NA	AWD, 3Y
		68/M	Oral tongue	NA	NA	NA	NA	II	NA	AWD, 1Y
		62/M	Oral tongue	NA	NA	NA	NA	IV	NA	NED, 2Y
		39/M	Oral tongue	NA	NA	NA	NA	II	NA	AWD, 18M
		44/F	Oral tongue	NA	NA	NA	NA	III	NA	NED, 3Y
Santhosh et al. [25]	2022	48/F	Oral tongue	NA	－	NA	NA	NA	NA	NA
Jain et al. [26]	2023	32/F	Base of the tongue	NA	＋	BSCC	NA	NA	NA	NA
Khan et al. [8]	2023	70/M	Oral tongue	＋	＋	BSCC	T4aN2bM0	IVA	CCRT	NA
Present case	2025	79/M	Base of the tongue	＋	－	BSCC	T2N1M0	III	CT + S with ND	NED, 23M

Oral candidiasis is a fungal infection of the oral cavity, and *Candida albicans* is the most common causative species, although *Candida glabrata* and *Candida tropicalis* have also been detected. The toxins produced are weak, and growth is normally suppressed by the immune system of the oral mucosa; however, when the immune system is weakened due to aging or comorbidities, fungi proliferate and oral candidiasis develops. In most cases, fungal growth is alleviated by the administration of antifungal agents. However, in immunocompromised or easily infected patients, *Candida *fungi can perforate the mucosal epithelium and damage it via extracellular enzymes, resulting in intractable and chronic candidiasis. Chronic candidiasis is associated with oral cancer and consists of two types: hypertrophic and atrophic. CHC, characterized by proliferative changes in the epithelial tissue, is considered to be associated with a higher risk of carcinogenesis; Bartie et al. reported that dysplasia occurred in 15% of CHC cases, with oral squamous cell carcinoma (OSCC) developing in 10% of the patients with CHC [30]. In 2022, Hsieh et al. reported that the interaction between cancer cells and the tumor microenvironment is important for the progression of OSCC and that *Candida albicans*, which is frequently present in oculopharyngeal muscular dystrophy (OPMD) and OSCC tissues, promotes malignant transformation [31]. Several hypotheses have been proposed as a mechanism for this: 1) *Candida albicans* infection promotes OSCC development by increasing tumor-associated macrophage infiltration [32]; 2) extracellular lipids produced by *Candida albicans* have been shown to enhance cellular metabolism and directly interact with dysplastic and malignant oral epithelial cells [33]; 3) the KRAS signaling pathway and E2F target downstream genes are involved in OSCC carcinogenesis caused by *Candida albicans* infection, which is different from the mechanism of OSCC carcinogenesis without *Candida albicans* infection, suggesting that *Candida albicans* is highly involved in malignant transformation [31]; 4) *Candida *species produce carcinogens such as nitrosamine compounds and acetaldehyde, with nitrosamine compounds activating specific oncogenes that cause malignant transformation [34,35]; and 5) acetaldehyde mutates DNA and induces the development of oral cancer, and alcohol consumption has been reported to promote the upregulation of acetaldehyde metabolism in *Candida *[36]. It has also been suggested that diabetes-related hyperglycemia is involved in promoting tumor progression in OPMDs [37]. In this case, *Candida albicans *had been confirmed by bacteriological examinations since 2007, and a biopsy in 2020 showed "intraepithelial *Candida *infiltration," suggesting that chronic inflammatory changes caused by *Candida albicans* and the susceptibility to infection due to type 2 diabetes mellitus may have contributed to the transformation to atypical epithelium. The patient had been on oral antidiabetic therapy for at least 15 years, from 2007 to 2022, and was presumed to have been chronically immunocompromised due to long-standing diabetes. In addition, the increased production of carcinogenic substances due to long-term alcohol consumption may have contributed to malignant transformation.

BSCC is thought to originate from cells in the basal cell layer of the surface epithelium or in the proximal tubules of secretory glands [26]; however, its pathogenesis is not fully understood. In addition, there have been no reports of BSCC, which is thought to have been caused by oral candidiasis, including CHC. The exact mechanism by which oral candidiasis causes BSCC remains unclear, and the further accumulation of cases and elucidation of its pathogenesis are expected in the future. In any case, patients with long-standing CHC are at high risk for carcinogenesis and should be followed up at short intervals, always paying attention to the possibility of malignant transformation.

As there is no standard treatment protocol for BSCC, it is presently treated according to the treatment for SCC. However, it is necessary to consider the high possibility of early, extensive invasion and metastasis. BSCC is associated with an extremely high rate of cervical lymph node metastasis (>60%) and distant metastasis to the lung, bone, brain, and skin (44%) [38], and the rate of distant metastasis is four to six times higher than that of SCC [39]. Therefore, radical resection with a sufficient safety margin is considered important for treatment [9, 38, 40, 41], and given the high rate of metastasis, more aggressive prophylactic/therapeutic neck dissection should be considered relative to conventional SCC. As for chemotherapy, no consensus has been reached because BSCC is a rare disease, but it is considered to be relatively sensitive to radiotherapy [9]. In this case, the patient was diagnosed with poorly differentiated SCC based on a biopsy. Because of rapid progression and cervical lymph node metastasis within a few months, we performed hemiglossectomy and neck dissection as pull-through surgery after PCE therapy as ICT. In addition, since patients with BSCC often have distant metastasis, postoperative chemoradiotherapy was planned; however, the patient did not consent to it. Among the 33 BSCC cases mentioned above, surgical resection was performed as the primary treatment in 17 (89.4%) of the 19 cases in which treatment was described. Concurrent neck dissection was performed in 12 of the 17 patients (70.5%), and neoadjuvant and adjuvant therapy was performed in five patients (29.4%). As for non-surgical treatment, chemoradiotherapy was used in one case, and chemotherapy alone was used in one case. Regarding the prognosis, nine of the 23 patients experienced recurrence or metastasis. The mean survival time of the five patients who died of recurrence or metastasis after treatment was 13.2 months, suggesting that they are associated with early progression and death. Therefore, for the early detection of recurrence and metastasis, systemic examinations at shorter intervals (relative to SCC) are considered necessary. Intraoral examinations once a month for the first year after surgery and imaging examinations combining various modalities (e.g., ultrasound, CT, MRI, and PET) every few months are recommended. The patient is currently doing well with no recurrence or metastasis; however, further careful follow-up is planned.

## Conclusions

To the best of our knowledge, this is the first report of a case of BSCC following tongue CHC. In this case, chronic inflammation caused by CHC, chronic alcohol consumption, and diabetes mellitus may have been associated with the development of BSCC. Given that CHC sometimes influences the development of malignant diseases, close follow-up is necessary. We emphasize the need to document and evaluate future cases to improve the treatment strategies and outcomes of this disease.
